# Characterization of complete mitochondrial genome of rice flower carp *Cyprinus carpio rubrofuscus* (Cypriniformes: Cyprinidae)

**DOI:** 10.1080/23802359.2021.1970642

**Published:** 2021-09-13

**Authors:** Manfen Lin, Yao Li, Yuehong Luo, Jinhui Chen, Xiaoling Qin

**Affiliations:** a Qingyuan Polytechnic, Qingyuan, China; b Guangdong Polytechnic of Science and Trade, Guangzhou, China

**Keywords:** *Cyprinus carpio rubrofuscus*, subspecies, mitochondrial genome, phylogeny

## Abstract

Rice flower carp (*Cyprinus carpio rubrofuscus*) is a subspecies of *Cyprinus carpio* distributed in South China. In this study, we sequenced the mitochondrial genome of *C. carpio rubrofuscus* using Illumina HiSeq. The complete mitochondrial genome of *C. carpio rubrofuscus* was 16,582 bp in length, including 13 protein-coding genes, 22 transfer RNA genes, and two ribosomal RNA genes. The contents of the four bases in the mitochondrial DNA were A (31.89%), T (24.82%), C (27.53%), and G (15.76%). Phylogenetic analysis showed that *C. carpio rubrofuscus* was clustered with *Cyprinus carpio* and its varieties.

Rice flower carp (*Cyprinus carpio rubrofuscus*) is a subspecies of *Cyprinus carpio* found widely in South China, such as the Pearl River and Hainan Island regions, where it inhabits the grassy or muddy edges of rivers (Wu 1977). The species is also commonly cultured with rice crops in western and northern Guangdong, China (Wu 1977). The body of *C. carpio rubrofuscus* varies from striped and flat to round and thick and its color varies from steel and light gray to golden red. The species is not large (∼50–250 g) and is characterized by cold-resistance, alkali-resistance, and hypoxic tolerance (Ma et al. 2018). However, research on this species remains limited. Ma et al. (2018, 2019) studied the morphological characteristics and genetic diversity of *C. carpio rubrofuscus*. The complete mitochondrial genome (mitogenome) of *C. carpio rubrofuscus* is an important resource for evolutionary research. In this study, we report on the complete mitogenome of *C. carpio rubrofuscus* and analyze its phylogenetic relationships within *Cyprinus*.

Specimens (voucher no. QP20190316-9) were collected from the Beijiang River (23°19′ N, 113°16′ E), Qingyuan, Guangdong, China, and were stored in the herbarium of Qingyuan Polytechnic (Guangdong, China). Muscle samples of *C. carpio rubrofuscus* were dissected and preserved at −80 °C until use. The muscle tissue was used for genomic DNA extraction with an E.Z.N.A.^®^ Tissue DNA Kit (Omega Bio-Tek, Guangzhou, China) following the manufacturer’s specifications. The mtDNA was sequenced using Illumina HiSeq (Illumina Inc., San Diego, CA). Clean data were acquired and assembled using SPAdes v3.15.2 (Bankevich et al. 2012) and PRICE (Ruby et al. 2013). MITO (http://mitos.bioinf.uni-leipzig.de/index.py) (Bernt et al. 2013) and ORF finder (https://www.ncbi.nlm.nih.gov/orffinder/) were used to identify and annotate protein-coding, transfer RNA (tRNA), and ribosomal RNA (rRNA) genes. Phylogenetic analysis was conducted using maximum-likelihood (ML) in MEGA X (Kumar et al. 2018).

The mitogenome of *C. carpio rubrofuscus* was 16,582 bp in length (GenBank accession number: MW969691) and contained 13 protein-coding, 22 tRNA, and two rRNA genes. Of the 37 genes, 28 were encoded by the heavy strand and nine, including one protein-coding (*ND6*) gene and eight tRNA genes, were encoded by the light strand. The A, G, C, and T contents of the heavy strand were 31.89%, 15.76%, 27.53%, and 24.82%, respectively, with a high C + G content of 43.29%. Most protein-coding genes had an ATG start codon, except *Cox1* (initiated with GTG). Seven protein-coding genes (*ND1*, *Cox1*, *ATP6*, *Cox3*, *ND4L*, *ND5*, and *ND6*) contained a TAA stop codon, three protein-coding genes (*ND2*, *ND3*, and *ATP8*) contained a TAG stop codon, and three protein-coding genes (*Cytb*, *ND4*, and *Cox2*) contained an incomplete T– stop codon. 16S rRNA and 12S rRNA were 1679 bp (56.40% AT content) and 953 bp (51.42% AT content) in length, respectively. All tRNA genes ranged from 67 to 76 bp in size.

A phylogenetic tree was constructed to validate the phylogenetic position of *C. carpio rubrofuscus.* The complete concatenated protein sequence which was coded by 13 protein-coding genes of another 22 *Cyprinidae* varieties was downloaded from GenBank. A phylogenetic tree was constructed using the ML method and Jones–Taylor–Thornton (JTT) matrix-based model, with a bootstrap of 1000 replicates (Figure 1). The phylogenetic tree showed that all *Cyprinus carpio* individuals and subspecies were clustered together. In conclusion, our study described the complete mitogenome of *C. carpio rubrofuscus* and analyzed its phylogenetic position within the *Cyprinus* genus. This research should contribute to further investigations on the molecular evolution and conservation of this species.

**Figure 1.**
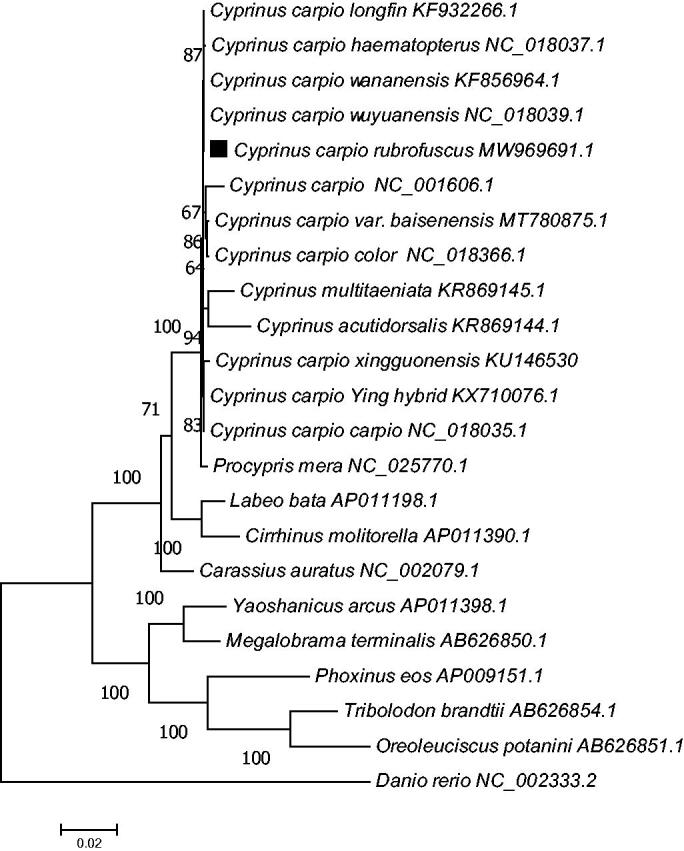
Phylogenetic tree of *C. carpio rubrofuscus* and related species based on maximum-likelihood (ML) method.

## Data Availability

Mitogenome data supporting this study are openly available in GenBank at: https://www.ncbi.nlm.nih.gov/nuccore/MW969691.1/. Associated BioProject, SRA, and BioSample accession numbers are http://www.ncbi.nlm.nih.gov/bioproject/742061, https://www.ncbi.nlm.nih.gov/sra/PRJNA742061, and https://www.ncbi.nlm.nih.gov/biosample/SAMN19923788, respectively.
